# The time revolution in macromolecular crystallography

**DOI:** 10.1063/4.0000247

**Published:** 2024-04-12

**Authors:** Georgii Khusainov, Joerg Standfuss, Tobias Weinert

**Affiliations:** Laboratory of Biomolecular Research, Division of Biology and Chemistry, Paul Scherrer Institut, Villigen PSI, Switzerland

## Abstract

Macromolecular crystallography has historically provided the atomic structures of proteins fundamental to cellular functions. However, the advent of cryo-electron microscopy for structure determination of large and increasingly smaller and flexible proteins signaled a paradigm shift in structural biology. The extensive structural and sequence data from crystallography and advanced sequencing techniques have been pivotal for training computational models for accurate structure prediction, unveiling the general fold of most proteins. Here, we present a perspective on the rise of time-resolved crystallography as the new frontier of macromolecular structure determination. We trace the evolution from the pioneering time-resolved crystallography methods to modern serial crystallography, highlighting the synergy between rapid detection technologies and state-of-the-art x-ray sources. These innovations are redefining our exploration of protein dynamics, with high-resolution crystallography uniquely positioned to elucidate rapid dynamic processes at ambient temperatures, thus deepening our understanding of protein functionality. We propose that the integration of dynamic structural data with machine learning advancements will unlock predictive capabilities for protein kinetics, revolutionizing dynamics like macromolecular crystallography revolutionized structural biology.

## INTRODUCTION: TRANSITION TO DYNAMIC CRYSTALLOGRAPHY

Macromolecular crystallography has been essential for determining the atomic structures of a vast array of proteins that are central to numerous cellular functions. However, crystallography's dominance in unveiling new structures is waning due to three main factors. First, most proteins suitable for x-ray structure determination have already been characterized. Second, single particle cryo-electron microscopy (cryo-EM) has advanced considerably in determining the structures of relatively rigid and large proteins, filling the gaps in our structural knowledge. In addition, cryo-EM is progressively enabling the study of more flexible and smaller proteins. This trend is evident in the decreasing number of x-ray structure-related articles and increasing cryo-EM structure-related publications in high-impact journals. Third, the collective triumphs of structural biology in protein structure determination, augmented by sequence data and advances in machine learning, have spurred the development of highly accurate structure prediction methods.^1,2^ Consequently, there is a movement from mere structure determination to a more functional approach, employing diverse methods to decipher protein function at the atomic scale.

The most critical aspect of protein function is their dynamic nature that allows for specific conformational rearrangements to drive essential biological processes such as catalysis, energy conservation, ion transport, signal transduction, and regulation. Our understanding of the structural evolution of reaction state intermediates has significantly advanced through the use of low temperatures to slow down catalytic rates in enzymes, capturing transition states.^3^ Freeze-trapping approaches have also been employed early on to study photo-cycle intermediates of the prototypical proton pump bacteriorhodopsin^4^ and many other photoactive proteins. There are new developments promising very fast freeze-trapping experiments, potentially re-invigorating the method.^5^ Nevertheless, the pursuit of truly time-resolved experiments with atomic resolution for studying protein dynamics has been of immense interest since the Myoglobin structure^6^ revealed that a static structure alone cannot fully explain protein function.^7^ To accurately study transitions between conformations, it is vital to conduct experiments at ambient temperatures where physiological changes are possible in contrast to when the protein is frozen in place. Time-resolved structural biology aims at understanding how proteins change their structure over time and how these conformational changes are related to protein function.

Currently, high-resolution crystallographic studies stand alone in their capacity to explore rapid dynamic processes in atomic detail. Now it is the time to delve deeper into the functional aspects of protein structures, some of which may have been determined more for cataloging purposes or to solve the phase problem^8^ rather than a desire to unravel their mechanistic secrets. The techniques and technology discussed here are gradually expanding the possibilities for dynamic studies, potentially elevating dynamic crystallography from the niche status it occupied since first pioneering experiments allowed us to study conformational changes in proteins over time.^9–11^ This perspective underscores the opportunities in dynamic crystallography, spurred by advancements in next-generation synchrotron and x-ray free electron laser (XFEL) facilities, even as static structure elucidation continues to accelerate.

## A BRIEF HISTORY OF TIME-RESOLVED CRYSTALLOGRAPHY: FROM LAUE TO XFELs

**FIG. 1.**
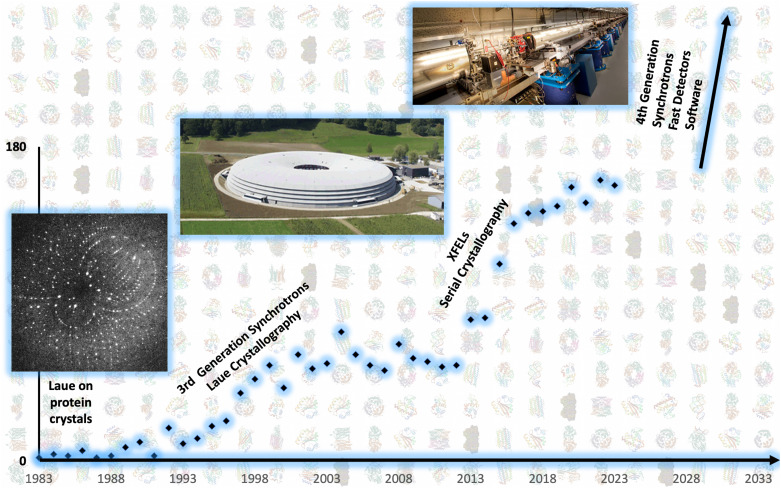
Evolution and milestones in time-resolved crystallography. Graph shows Google Scholar results per year using the term “time-resolved crystallography.” Key historical developments are annotated on the timeline, including from left to right: the publication of the first Laue diffraction pattern from protein crystals (1984)^13^ [picture from Fig. 2(b) reprinted with permission from Moffat *et al.*, Science **223**(4643), 1423–1425 (1984). Copyright 1984 AAAS], the inauguration of the Swiss Light Source (SLS) (2001)^14^ (Picture: SLS), and the Linac Coherent Light Source (LCLS) tunnel (2009) upon first lasing^15^ (picture: Brad Plummer/SLAC National Accelerator Laboratory). The backdrop displays all time-resolved structures archived in the Protein Data Bank (PDB). The inception of time-resolved crystallography coincides with the advent of Laue crystallography's rapid data acquisition capabilities. Its initial growth phase was bolstered by the launch of third-generation synchrotrons between 1994 (ESRF) and 2016 (SESAME). Time-resolved crystallography grew further with the emergence of x-ray free-electron lasers (XFELs) between 2009 and 2018, further bolstered by serial crystallography techniques. The field is expected to gain additional momentum over the next decade, propelled by advancements in high-speed detectors, fourth-generation synchrotron sources, and novel computational methods, transcending its niche origins to broaden its impact and research output.

The first time-resolved crystallographic (TRX) study at physiological temperatures, conducted in 1987,^12^ demonstrated the phosphorylation of heptenitol by glycogen phosphorylase b over approximately 2 h. The study involved soaking crystals in substrates and collecting data on film in as little as 25 min per dataset. At that time, macromolecular crystallographic data collection primarily relied on film, but faster data acquisition rates were achievable using the Laue approach, first successfully implemented for protein crystals in 1984.^13^ The Laue method, which did not require crystal rotation, facilitated the rapid capture of complete datasets using a broad x-ray spectrum. Laue crystallography was the spark that initiated an ever-increasing interest in TRX (Fig. 1).

With this technique, the speed of data acquisition ceased to be a limiting factor for well-ordered crystals. First Laue difference maps demonstrated that Laue could be used for fast data collection,^16^ quickly followed in 1990 by the first time-resolved study, which determined the structure of the small G-protein Ha-Ras p21 in its unstable guanosine triphosphate (GTP) complex generated 4 min after photo-uncaging of GTP and followed its partial hydrolysis to GDP over 14 min.^17^ That same year, a time-resolved study on Chymotrypsin^18^ showcased an alternative approach, using electronic detection on a four-circle diffractometer to observe photolysis of an inhibitor directly, a task not possible with film. This, however, came with the trade-off of not being able to capture time-resolved data for the complete structure, an issue that was later addressed in a subsequent Laue study.^19^ In 1992, a flow-cell setup enabled the real-time creation of an uninhibited form of Trypsin via a pH-Jump,^20^ and the structure was determined minutes after pH-induced deacylation.^21^

Subsequent studies in time-resolved crystallography began targeting even faster processes. In 1996, the photolysis of the carbon monoxide (CO)–Myoglobin complex was observed with nanosecond time-resolution using a CCD detector,^22^ and the first intermediate state of a photoreceptor, photoactive yellow protein (PYP), was captured with millisecond time-resolution in 1997 using an image plate detector.^23^ The time-resolution of synchrotron radiation is inherently limited to about 150 ps by the x-ray pulse generated from a single electron bunch, a threshold that was reached in 2003 for the Myoglobin system, also employing a CCD detector.^24^ However, it is noteworthy that only by accumulating multiple picosecond pulses was the data interpretable, thus severely restricting the range of accessible systems. That changed with the emergence of XFELs, producing bright, indexable, and nearly monochromatic diffraction patterns within femtosecond exposures. Both Myoglobin^25^ and PYP^26,27^ have been at the forefront of the XFEL revolution in TRX, pushing timing boundaries even further into the femtosecond range. Today, a multitude of new systems have been examined, yielding novel insights into a broad spectrum of biological reactions. Table I summarizes TRX experiments with deposited coordinates in the wwPDB. For studies conducted prior to wwPDB depositions were standard, the comprehensive review by Hajdu and Andersson from 1993 provides an excellent overview.^11^

**TABLE I.** List of time-resolved experiments. The list was curated manually by searching the PDB for all structures deposited above the glass transition (<180 K) and, hence, may not be complete. Single shot serial crystallography experiments (ssSX) are highlighted in green.

Corresponding authors and last author of primary citation	Publication year	PDB code^a^	Target	X-ray source	Time-resolved crystallography method	Activation	Time-scale
Gouet and Hajdu^46^	1996	2CAG	Catalase	Photon factory (BL-6A2)	Rotation (Weissenberg camera)	Mixing (flow cell)	min
Getzoff^23^	1997	2PYP	PYP	NSLS (X26C)	Laue	CW-laser	ms, photo-stationary
Helliwell and Hadener^47^	1998	1YPN	Hydroxymethylbilane synthase	ESRF (ID09)	Laue	Mixing (flow cell)	min, h
Moffat^48^	1998	2PYR	PYP	ESRF (ID09)	Laue	ns-laser	ns
Zegers and Wyns^49^	1998	1GSP, 3GSP, 4GSP, 6GSP	RNase T1	Home source	Rotation	Mixing (soaking)	days, weeks
Bourgeois and Brunori^50^	2003	1MZ0	Myoglobin (Heme-CO)	ESRF (ID09)	Laue	ns-laser	ns, *μ*s, ms
Norris^51^	2004		Photosynthetic reaction center	APS (BioCARS)	Laue	ns-laser	(ms)
Andersonand Moffat^52^	2004		PYP	APS (BioCARS), ESRF (ID09)	Laue	ns-laser	ns, *μ*s, ms
Schmidt and Moffat^53^	2004	1S4S, 1S4R	PYP	APS (BioCARS)	Laue	ns-laser	*μ*s, ms
Schmidt and Šrajer^54^	2005	2BWH	Myoglobin (Heme-CO)	APS (BioCARS)	Laue	ns-laser	(ns, ms, s), *μ*s
Moffat^55^	2005	1T18, 1T19, 1T1A, 1T1B, 1T1C	PYP	APS (BioCARS), ESRF (ID09)	Laue	ns-laser	ns, *μ*s, ms
Ihee and Moffat^56^	2005	1TS8, 1TS7, 1TS6, 1TS0	PYP	APS (BioCARS), ESRF (ID09)	Laue	ns-laser	ns, *μ*s, ms, s
Phillips, Jr.^57^	2006	2G0V, 2G0X, 2G0Z, 2G10, 2G11, 2G12, 2G14	Myoglobin (heme-CO)	ESRF (ID09)	Laue	fs-laser	ps, ns, *μ*s
Šrajer and Royer, Jr.^58^	2006	2GRZ	Hemoglobin (heme-CO)	APS (BioCARS)	Laue	ns-laser	ns (*μ*s)
Moffat^59^	2007	2OWH	FixL (heme-CO)	APS (BioCARS)	Laue	ns-laser	*μ*s (ms)
Šrajer and Royer, Jr.^60^	2009		Hemoglobin (heme-CO)	APS (BioCARS)	Laue	ns-laser	(ns, *μ*s)
Neutze^61^	2010	2X5V	Photosynthetic reaction center	ESRF (ID09)	Laue	ns-laser	ms
Schmidt^62^	2012	3UMD, 3UME	PYP	APS (BioCARS)	Laue	ns-laser	*μ*s, ms (s)
Ren and Royer, Jr.^63^	2012	3QOB	Hemoglobin (heme-CO)	APS (BioCARS)	Laue	fs-laser	ps
Anfinrud^64^	2012	4B9O, 4BBT, 4BBU, 4BBV	PYP	APS (BioCARS)	Laue	fs-laser	ps, ns, *μ*s, ms
Ihee^65^	2013	3VE3, 3VE4, 4HY8, 4I38, 4I39, 4I3A, 4I3I, 4I3J	PYP	APS (BioCARS), ESRF (ID09)	Laue	fs-laser	ps, ns, *μ*s
Yachandra, Bergmann, and Yano^66^	2013	4IXR	Photosystem II	LCLS (CXI)	(FEL)-ssSX (electrospinning injector)	ns-laser	ms
Mizutani and Suzuki^67^	2014	3WU7, 3WPK, 3WU8, 3WU9, 3WPL, 3WUA	Lysozyme	Spring-8 (BL26B2)	Rotation	Mixing (soaking)	min, h
Fromme^68^	2014	4PBU, 4RVY	Photosystem II	LCLS (CXI)	(FEL)-ssSX (Gas virtual dynamic nozzle [GVDN])	Laser diode	*μ*s
Schmidt^27^	2014	4WLA	PYP	LCLS (CXI)	(FEL)-ssSX (GVDN)	ns-laser	(ns) *μ*s
Barends and Schlichting^25^	2015	5CN4, 5CN5, 5CN6, 5CN7, 5CN8, 5CN9, 5CNB, 5CNC, 5CND, 5CNE, 5CNF, 5CNG	Myoglobin	LCLS (CXI)	(FEL)-ssSX (GVDN)	fs-laser	fs, ps
Standfuss^69^	2016		Bacteriorhodopsin	LCLS (CXI)	(FEL)-ssSX (high viscosity injector)	fs-laser	(ms)
Perbandt^70^	2016	5LH0, 5LH1, 5LN0, 5LH3, 5LH5, 5LMH; 5LH6, 5LH7	Thaumatin	P14 (PETRA III)	Rotation-SSX	x-ray (radiation damage)	ms
Schmidt^26^	2016	5HDC, 5HDD, 5HDS, 5HD5	PYP	LCLS (CXI)	(FEL)-ssSX (GVDN)	fs-laser	fs, ps
Ranganathan^71^	2016	5E22	PDZ domain	APS (BioCARS)	Laue	Electric field	ns
Neutze and Iwata^43^	2016	5B6W, 5H2H, 5H2I, 5H2J, 5B6X, 5H2K, 5H2L, 5H2M, 5B6Y, 5H2N, 5H2O, 5H2 P, 5B6Z	Bacteriorhodopsin	SACLA (BL3)	(FEL)-ssSX (high viscosity injector)	ns-laser	ns, *μ*s, ms
Yoshikawa and Tsukihara^72^	2017	5X1B, 5X19	Cytochrome C oxidase	SACLA (BL3)	(FEL)-ssSX (loop)	ns-laser	ns, *μ*s
Iwata and Shen^73^	2017	5WS5, 5GTI	Photosystem II	SACLA (BL3)	TR-(FEL)-ssSX (high viscosity injector)	ns-laser	ms
Wang^74^	2017	5SWD, 5SWE	Adenine riboswitch	LCLS (CXI)	(FEL)-ssSX (GVDN)	Mixing (T-junction)	s, min
Sugimoto, Shiro, and Kubo^75^	2017	5Y5K	NO reductase	SACLA (BL3)	TR-(FEL)-ssSX (high viscosity injector)	ns-laser	ms
Pai and Miller^76^	2018	6GXH, 6GXD, 6GXT	Fluoroacetate dehalogenase	PETRA III (P14)	(Synchrotron)-ssSX (Fixed Target)	fs-laser (photocage)	ms, s
Standfuss^77^	2018	6G7I, 6G7J, 6G7K, 6G7L	Bacteriorhodopsin	LCLS (CXI)	(FEL)-ssSX (high viscosity injector)	fs-laser	fs, ps, ms
Colletier, Schlichting, and Weik^78^	2018	5O8B, 5O8C	Green fluorescent protein (GFP)	LCLS (CXI)	(FEL)-ssSX (GVDN)	fs-laser	ps
Schmidt^79^	2018	6B5Y, 6B68, 6B69, 6B6A, 6B6C, 6B6D, 6B6E, 6B6F	β-Lactamase	LCLS (CXI)	(FEL)-ssSX (GVDN)	Mixing (nozzle)	ms, s
Domratcheva and Schlichting^80^	07/2019	6GA3, 6GA4, 6GA5, 6GA6, 6GA7, 6GA8, 6GA9, 6GAA, 6GAB, 6GAC, 6GAD, 6GAE, 6GAF, 6GAG, 6GAH, 6GAI	Bacteriorhodopsin	LCLS (CXI)	(FEL)-ssSX (high viscosity injector)	fs-laser	fs, ps, ms
Weinert and Standfuss^81^	07/2019	6RNJ, 6RPH, 6RQO	Bacteriorhodopsin	SLS (X06SA)	(Synchrotron)-ssSX (high viscosity injector)	Laser diode	ms, photostationary
Miller and Pai^82^	09/2019	6QHY, 6QHV, 6QHU, 6QHT, 6QHS, 6QHQ, 6QHP, 6QHW, 6QHX, 6QHZ, 6QI0, 6QI1, 6QI2, 6QI3	Fluoroacetate dehalogenase	PETRA III (P14)	(Synchrotron)-ssSX (Fixed Target)	fs-laser (photocage)	ms, s
Miller^83^	10/2019	6RNC, 6QNB, 6QNH, 6RND, 6RNF, 6QNC, 6QNI, 6QNJ, 6QND	Lysozyme, Xylose Isomerase	PETRA III (P14)	(Synchrotron)-ssSX (Fixed Target)	Mixing (droplet application)	ms, s
van den Bedem and Wilson^84^	11/2019	6UND, 6UNF	Isocyanide hydratase	LCLS (MFX)	(FEL)-ssSX (coMESH injector)	Mixing (T-junction)	s, min
Schmidt^85^	01/2020	6P4I, 6P5D, 6P5E	PYP	EuXFEL [SPB/(FEL)-ssSX]	(FEL)-ssSX (GVDN)	fs-laser	ps, *μ*s
Sliwa, Schlichting, and Weik^86^	02/2020	6T3A	GFP	SACLA (BL3)	(FEL)-ssSX (GVDN)	fs-laser	ns
Schmidt and Westenhoff^87^	03/2020	6T3U	Bacterial phytochrome	SACLA (not mentioned)	(FEL)-ssSX (high viscosity injector)	fs-laser	ps
Quiney, Ziaja, and Schlichting^88^	04/2020	6SRQ, 6SRK, 6SRL, 6SRO, 6SRP, 6SR1, 6SR2, 6SR3, 6SR4, 6SR5	Ferredoxin	LCLS (CXI)	(FEL)-ssSX (GVDN)	X-ray (radiation damage)	fs
Standfuss^89^	05/2020	6TK1, 6TK2, 6TK3, 6TK4, 6TK5, 6TK6, 6TK7	Sodium pumping rhodopsin	SwissFEL (ALVRA)	(FEL)-ssSX (high viscosity injector)	fs-laser	fs, ps, ns, ms
Messinger, Yachandra, and Yano^90^	06/2020	6W1P, 6W1Q, 6W1R, 6W1T, 6W1U, 6W1V	Photosystem II	LCLS (MFX), SACLA (BL2)	(FEL)-ssSX (drop on tape)	ns-laser	*μ*s, ms
Royant^91^	07/2020	6S46	LOV2 domain of phototropin-2 from *Arabidopsis thaliana*	ESRF ID30A-3	TR-SOX (serial oscillation crystallography)	LED	ms
Neutze^92^	01/2021	6ZHW, 6ZI4, 6ZI5, 6ZI6, 6ZI9, 6ZIA, 6ZID	Bacterial photosynthetic reaction center	LCLS (CXI)	(FEL)-ssSX (GVDN)	fs-laser	ps, *μ*s
Wang^93^	03/2021	6VWT, 6VWV	Adenine riboswitch	LCLS (CXI)	(FEL)-ssSX (GVDN)	Mixing (T-junction)	s, min
Stan and Schlichting^94^	03/2021	7AEV	Hemoglobin α-subunit	LCLS (CXI)	(FEL)-ssSX (GDVN)	fs-laser (pump x-ray pulse)	ns
Schmidt, Lee, and Liu^95^	03/2021	7CRI, 7CRK, 7CRL, 7CRS, 7CRT, 7CRX, 7CRY	Chloride pumping rhodopsin	LCLS (CXI)	(FEL)-ssSX (high viscosity injector)	fs-laser	ps
Kubo, Nishizawa, and Nureki^96^	03/2021	7E6X, 7E6Y, 7E6Z, 7E70, 7E71	Channelrhodopsin	SACLA (BL3)	(FEL)-ssSX (high viscosity injector)	ns-laser	*μ*s, ms
Shen and Suga^97^	04/2021	7CJI, 7CJJ	Photosystem II	SACLA (BL3)	(FEL)-ssSX (high viscosity injector)	ns-laser	ms
Berthomieu *et al.*^98^	04/2021		Fatty acid photodecarboxylase	LCLS (CXI)	(FEL)-ssSX (GVDN)	fs-laser	(ps, ns, *μ*s)^a^
Kern and Orville^99^	07/2021	7BH4, 7BH5, 7BH7, 7BHL, 7BHM, 7BHN	Lysozyme, β-lactamase	SACLA (BL2)	(FEL)-ssSX (drop on tape)	Mixing (drop on drop)	ms, s, min
Westenhoff, Stojković, and Schmidt^100^	07/2021	7JR5, 7JRI	Bacterial phytochrome	SACLA (BL2)	(FEL)-ssSX (high viscosity injector)	ns-laser	ns, ms
Kern, Orville, and Schofield^101^	08/2021	6ZAF, 6ZAG, 6ZAH, 6ZAI, 6ZAJ, 6ZAL	Isopenicillin N synthase	LCLS (MFX), SACLA (BL2)	(FEL)-ssSX (drop on tape)	Mixing (O_2_ atmosphere chamber)	ms, s
Schmidt^102^	08/2021	7K8E, 7K8F, 7K8H, 7K8K	β-Lactamase	EuXFEL (SPB/(FEL)-ssSX)	(FEL)-ssSX (GVDN)	Mixing (nozzle)	ms
Messinger *et al.*^103^	11/2021	7RF1, 7RF3, 7RF4, 7RF5, 7RF6, 7RF7, 7RF8	Photosystem II	LCLS (MFX)	(FEL)-ssSX (drop on tape)	ns-laser	*μ*s, ms
Nogly^104^	01/2022	7O8G, 7O8H, 7O8I, 708 J, 7O8K, 7O8M, 7O8N, 7O8O, 7O8P, 7O8Q, 7O8R, 7O8S, 7O8T, 7O8U, 7O8V	Chloride pumping rhodopsin	SLS (PXI) SwissFEL (Alvra)	(Synchrotron)-ssSX (FEL)-ssSX (high viscosity injector)	fs-laser, Laser diode	ps, ns, *μ*s, ms
Nango and Shirouzu^105^	01/2022	7VGU	Chloride pumping rhodopsin	SACLA (BL3)	(FEL)-ssSX (high viscosity injector)	ns-laser	ms
Essen, Bessho, and Tsai^106^	04/2022	7VIX, 7VIY, 7VIZ, 7VJ0, 7VJ1, 7VJ2, 7VJ3, 7VJ4, 7VJ5, 7VJA, 7VJB, 7VJC, 7VJE, 7VJG, 7VJH, 7VJI, 7VJJ, 7VJK	DNA photolyase	SACLA (BL2)	(FEL)-ssSX (high viscosity injector)	ns-laser	ns, *μ*s, ms
Schlichting and Weik^107^	09/2022	7R33, 7R34, 7R35, 7R36	Fatty acid photodecarboxylase	LCLS (CXI)	(FEL)-ssSX (GVDN)	fs-laser	ps, ns, *μ*s
Royant^108^	09/2022	8A2W	Phototropin-2	ESRF (ID30A-3)	TR-SOX (Serial oscillation crystallography)	LED	min
Joachimiak^109^	11/2022	7UHT, 7UHH, 7UHI, 7UHJ, 7UHK, 7UHL, 7UHM, 7UHN, 7UHO, 7UHP, 7UHQ	Beta-lactamase type II	APS (14-ID-B)	(Synchrotron)-ssSX (fixed target)	ns-laser	ms, s
Steinmetz and Standfuss^110^	02/2023	7YYV, 7YYW, 7YYX, 7YYY, 7YYZ, 7YZ0, 7YZ1, 7YZ2, 7YZ5	Tubulin	SwissFEL (Alvra)	(FEL)-ssSX (high viscosity injector)	Laser diode	ns, *μ*s, ms
Schertler and Panneels^111^	03/2023	8A6C, 8A6D, 8A6E	Rhodopsin	SACLA (BL3_EH2) SwissFEL (Alvra)	(FEL)-ssSX (high viscosity injector)	fs-laser	ps
Gordeliy^112^	05/2023	7ZNE, 7ZNG, 7ZNH, 7ZNI	Xenorhodopsin	PETRA III (P14) ESRF (ID23-1)	(Synchrotron)-ssSX (high viscosity injector)	Laser diode	*μ*s, ms
Nango and van Thor^113^	07/2023	8A6N, 8A6O, 8A6P, 8A6Q, 8A6R, 8A6S	Green fluorescent protein	SACLA (BL3)	(FEL)-ssSX (droplet-on-demand)	fs-laser	fs, ps, *μ*s
van Thor^114^	08/2023	7QLN, 7QLO	rsKiiro	LCLS (CXI) SACLA (BL3) PAL-XFEL (NCI)	(FEL)-ssSX (GDVN)	fs-laser	ps
Schmidt^115^	09/2023	8EBI, 8EBR, 8EC4, 8ECF, 8GCS, 8GCT, 8GCX	β-lactamase	LCLS (MFX)	(FEL)-ssSX (mix-and-inject)	Mixing	ms, h
Nango and Thompson^116^	09/2023	8CVU, 8CVV, 8CVW, 8CV0, 8CV1, 8CW3, 8CW5, 8CW6, 8CW7, 8CWC, 8CWD, 8CWE, 8CWF, 8CWG, 8CWH	Lysozyme	SACLA (BL2)	(FEL)-ssSX (high viscosity injector)	Temperature	ns, *μ*s
Rousseau and Yeh^117^	09/2023	8GBT	Cytochrome c oxidase subunit 1	LCLS (CXI)	(FEL)-ssSX (GDVN)	ns-laser	ns
Kern and Liu^118^	11/2023	8TDP	Mycocyclosin synthase	LCLS (MFX)	(FEL)-ssSX (drop on tape)	Mixing	ms
Lane^119^	11/2023	8OY3, 8OY4, 8OY5, 8OY6, 8OY7, 8OY8, 8OY9, 8OYA, 8OYB, 8OYC	Deoxyribodipyrimidine photolyase	SwissFEL (Alvra)	(FEL)-ssSX (high viscosity injector)	fs-laser	ps, ns, *μ*s
Wranik, Kepa, and Standfuss^120^	12/2023	8CL8	Sodium pumping rhodopsin	SwissFEL (Alvra)	(FEL)-ssSX (high viscosity injector)	fs-laser	*μ*s
Maestre-Reyna *et al.*^121^	12/2023	7YCM, 7YCP, 7YCR, 7YD6, 7YD7, 7YD8, 7YEB, 7YEC, 7YEE, 7YEI, 7YEJ, 7YEK, 7YEL, 7YEM	Deoxyribodipyrimidine photolyase	SACLA (BL2) SwissFEL (Alvra)	(FEL)-ssSX (high viscosity injector)	ns-laser	ps, ns, *μ*s

^a^ Only PDB codes for activated structures or PDBs associated with time-resolved data are given (e.g., refinement of structural intermediate or time-resolved datasets deposited).

## PARALLEL PATHS: CRYO-CRYSTALLOGRAPHY'S RISE MIRRORS THE RISE OF CRYO-EM

**FIG. 2.**
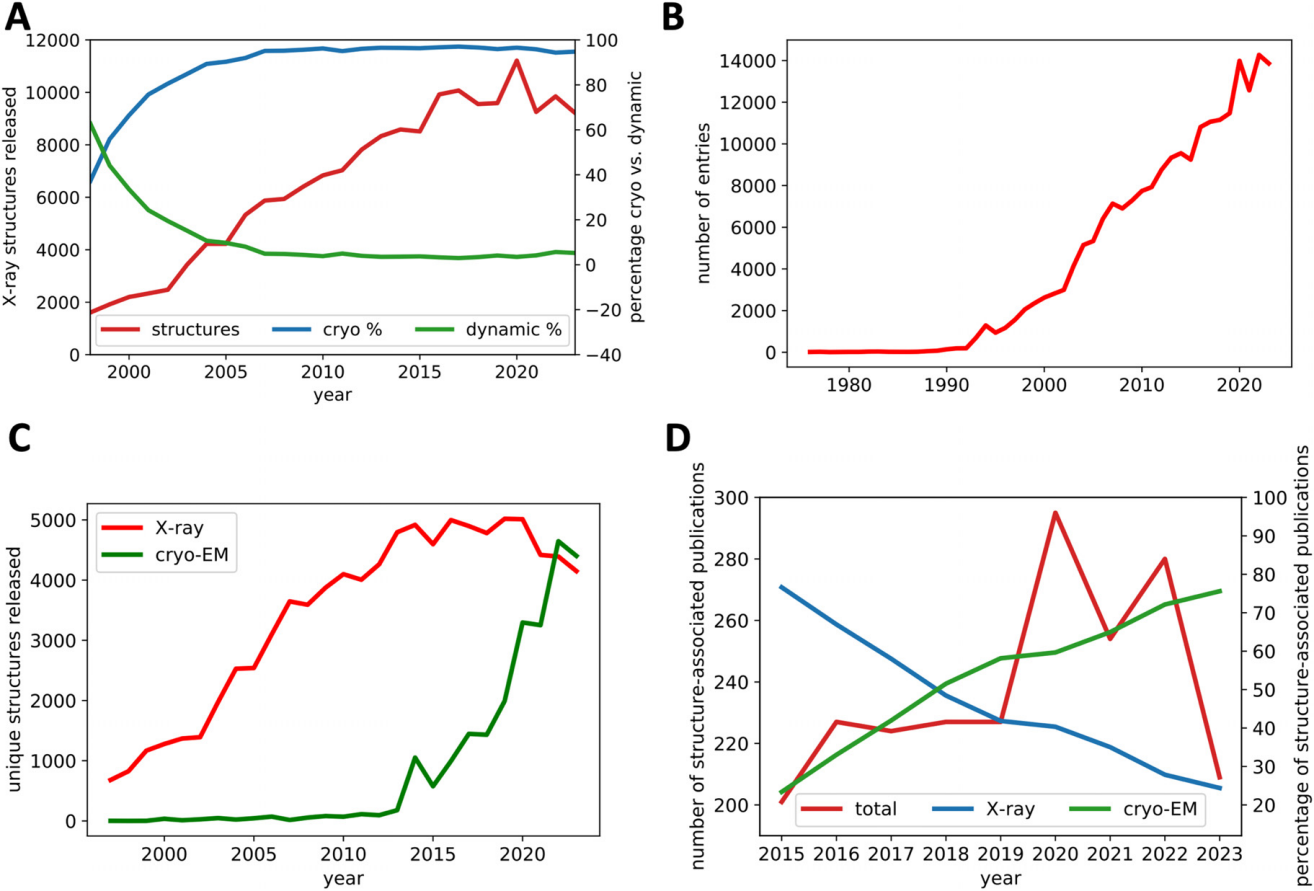
Growth trends in structural biology. (a) The total count of x-ray crystallography structures released annually by the Protein Data Bank (PDB) reached its peak in 2020 (red line). Starting in 1999, the release of cryo-structures (blue line) surpassed the number of “dynamic” structures here defined as temperatures above the glass transition (≥180 K) (green line). (b) The total number of PDB entries released annually continues to increase (red line), indicating an ongoing productivity increase in structural biology. (c) This upward trajectory is driven by cryo-EM (green line), which eclipsed the number of unique (molecules with the exact same sequence are counted just once) x-ray crystallography entries released by the PDB (red line) in 2022. (d) Anticipating this shift, the percentage of publications featuring cryo-EM structures (green line) outpaced those based on x-ray crystallography (blue line) in three high-impact journals (total number of publications shown as red line).

To fully understand the benefits of dynamic crystallography, it is essential to examine the historical context and developments that contributed to the success of cryo-crystallography (Fig. 2). Dynamic processes are time-dependent and require temperatures above the glass transition of water (<180 K) to occur. Nevertheless, most of all structural information on proteins has been obtained by studying individual structural states at cryogenic temperatures. Third-generation synchrotron sources facilitated the use of small crystals but increased radiation damage to samples, effectively making ambient temperature structure determination more challenging.^28^ Cryo-crystallography determined the structures of virtually all key players of life, ranging from the ribosome to respiratory and photosynthetic chains, to G protein-coupled receptor signaling complexes. With the onset of cryo-crystallography, structure determination at physiological temperatures fell out of favor, and surprisingly few systematic studies were conducted to examine the effects of cryo-cooling on crystal structures. Reviews on cryo-crystallography did not compare the effects of cryo-cooling on crystals on a structural level,^29–32^ and there were few systematic studies carried out.^33,34^ It was generally believed that cryo-crystallography had little effect on overall structures and led to more accurate and detailed models.^32^ These conclusions were drawn despite early evidence^35^ that temperature effects on protein structure could inhibit substrate binding. Consequently, a substrate binding site at cryogenic temperatures might not reflect this site in the biologically active enzyme.^35^ New methods had to be found to overcome the effects of radiation damage without compromising structure or function.

**FIG. 3.**
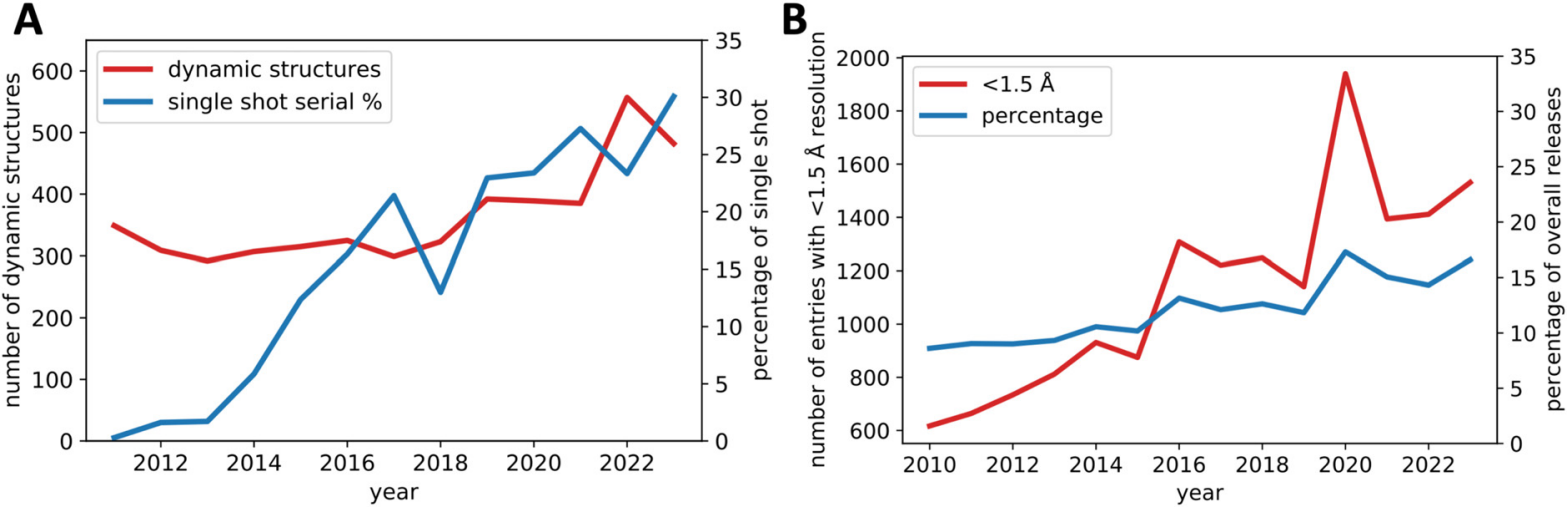
Rise in single-shot serial crystallography and high-resolution structural studies. (a) There is a noticeable uptick in “dynamic” structures, determined at temperatures over 180 K since 2018 (red line). Single-shot serial crystallography (ssSX) is becoming more prominent, making up 30% of these dynamic entries in the PDB by 2023 (blue line). (b) At the same time, high-resolution structures are claiming a larger share in the PDB. The red line shows the number of structures with resolutions higher than 1.5 Å, and the blue line indicates their percentage of total submissions. This trend highlights the crucial role of crystallography as a high-resolution method for in-depth structural insights.

Due to increased data quality and ease of use, cryo-crystallography had a major impact on the field. Over two decades, the number of released cryogenic structures (<180 K) in the wwPDB soared from 3488 in the year 2000 to 161 211 by the end of 2023. In stark contrast, the number of “dynamic” structures (≥180 K) lagged, increasing from 3752 to 12 657 [for an overview of annually released x-ray crystallographic structures and the percentage of structure below the glass transition and above, see Fig. 2(a)]. Here, we use the glass transition at 180 K to differentiate “dynamic” from cryogenic structures. Cryo-cooling best reduces radiation damage below the glass transition, which is crucial in cryo-crystallography. Protein motions initiate above this transition, which is exploited in multi-temperature crystallography.^36^ Starting from 1997 deposition rates kept increasing, but reached a plateau in 2014 and now, while the overall number of annually deposited structures still increases due to the rise of cryo-EM [Figs. 2(b) and 2(c)], the number of unique crystallographic structures released per year is declining [Fig. 2(c)]. This trend is not only reflected in depositions but also by a decrease in publications in high impact journals that are associated with x-ray crystallographic structures [Fig. 2(d)]. While there is growing interest in dynamic studies [Fig. 1, Table I, and Fig. 3(a)], the meteoric rise of cryo-EM placed static structure determination once more in the spotlight, mirroring the rise of cryo-crystallography and overshadowing the promising developments in dynamic studies. Given that accessing cryo-EMs is now less challenging than securing beamtime at fourth-generation synchrotron beamlines—or even more so at XFELs—and that structural determination experiments are simpler than those dissecting the dynamics, the structural dynamics field is in for fierce competition for both talent and recognition. Streamlining the workflow for time-resolved experiments and developing dedicated end stations are ongoing and seem more critical than ever. However, the time for initiating bold projects akin to the structural genomics initiatives^8^—which aimed to solve the phase problem and have seen fruition through artificial intelligence^1,2^—is now.

## SERIAL CRYSTALLOGRAPHY AND FAST DETECTORS: “SHOOT FIRST—ASK QUESTIONS LATER”

Serial crystallography (SX) addresses the issue of radiation damage by spreading the x-ray dose over a series of crystals. By exposing each crystal to the x-ray beam briefly and recording the diffraction pattern, and then aggregating the data from multiple crystals, a complete dataset is generated. In fact, many structural studies were based on serial crystallography prior to the rise of cryo-crystallography, even though this uses the term in a broader sense as is common today.^37^ It is almost forgotten that the term serial crystallography did not refer to a method centered around crystals in the first place.^38^ Similar to Laue crystallography, single shot serial crystallography (ssSX), which is discussed here, does not require crystal rotation. It exposes each region of a crystal only once to X-rays. Developments in detector technology as well as beamline instrumentation and new types of x-ray sources, while often long anticipated, have largely driven the development of time-resolved crystallography. However, the emergence of single-shot SX was not anticipated. It was born from the necessity to rapidly deliver fresh randomly oriented crystals for each XFEL pulse—i.e., “the American method of shooting first and asking questions later” (quoted in remembrance of John Spence).

The pioneering ssSX experiments utilized numerous nanocrystals at the Linac Coherent Light Source (LCLS) in Stanford, the first operational hard XFEL.^39^ From then on, ssSX at XFELs was producing structures at room temperature at fast pace, selected milestones were the first high-resolution structure,^40^ the first experimental phasing,^41^ the first novel G protein-coupled receptor structure,^42^ the first high-resolution time-resolved experiment,^27^ and the first time-resolved experiment resulting in a molecular movie of bacteriorhodopsin, a protein previously inaccessible to time-resolved crystallography.^43^ As ssSX matured, the focus of innovation shifted to sample delivery,^44,45^ with many techniques initially tailored for XFELs also proving beneficial at synchrotrons. Indeed, SX was posed to solve the major challenge of dynamic crystallography at physiological temperatures: radiation damage. Distributing the dose over many crystals in a synchrotron experiment is not as effective as the diffraction before destruction approach^122^ that makes XFEL structures damage free in most cases.^123^ Nevertheless, it allowed room temperature data collection on very small membrane protein crystals of bacteriorhodopsin^124^ and produced data of sufficient quality to allow *de novo* phasing with heavy atom derivatives of lysozyme^125^ at synchrotrons. Serial crystallography has facilitated routine room-temperature data collection beyond model systems,^126,127^ paving the way for a broader application of TRX, with ssSX constantly increasing its share of dynamic structures [Fig. 3(a)]. Along this path, the field will continue developing away from the study of well-diffracting model proteins toward the study of protein targets with the potential to reveal new fundamental insights through TRX studies on catalysis^79,99^ and even time-resolved studies on a cancer drug target.^110,120^ Another growth trend emphasizes that crystallography is moving from structure determination toward understanding function, as reflected by an ever-increasing fraction of high-resolution structures released by the wwPDB [Fig. 3(b)].

For TRX, rapid data acquisition is crucial. A long-standing aspiration in the field was to obtain a large-area detector capable of capturing and storing images faster than the lifespan of reactive enzyme intermediates.^9^ Laue crystallographers had long relied on film until the advent of larger CCD area detectors,^128^ but the necessary readout speeds for millisecond time-resolution were only achieved in 2003^129^ and first applied to protein studies using wide-angle x-ray scattering (WAXS) in 2010.^130^ While the Laue method would have provided the possibility to collect full crystallographic datasets using hybrid pixel detectors, the full potential of fast detectors was realized through ssSX to achieve biologically relevant time resolutions while enabling the study of many more systems.

Recent demonstrations show the effectiveness of this synergy, revealing the release of photocaged compounds,^76,82^ rapid ligand soaking,^83^ and photoactivation.^81^ These studies have uncovered details like enzyme cooperativity potentially mediated by a water molecule chain and significant conformational changes in bacteriorhodopsin during its photocycle. Other studies have employed rotation methods with high frame rate detectors for time-resolved experiments.^108^ The capabilities of detectors like EIGER^131^ and JUNGFRAU,^132^ with frame rates exceeding 2 kHz, have yet to be fully explored, potentially allowing sub-millisecond time resolutions without electronic gating or mathematical deconvolution methods like the Hadamard transform.^133^

Fast detectors not only facilitate megahertz-speed pulsed experiments at advanced light sources like the European XFEL^134,135^ but also enable rapid pump-probe experiments at fourth-generation synchrotrons using pink beams for maximal dose delivery in shorter time frames. The tremendous success of time-resolved XFEL experiments across various time scales (Table I) illustrates the efficacy of this classic pump-probe approach when combined with serial crystallography.

## ILLUMINATING DYNAMICS: FOURTH-GENERATION SYNCHROTRONS AND XFELs

XFELs hold an advantage over synchrotrons due to their ability to generate many diffracted photons per time from crystals at room temperature. Due to the diffraction-before-destruction principle, XFEL ssSX data often extend to higher resolutions than their synchrotron counterparts,^127^ especially when studying small membrane protein crystals.^136^ That said, when studying well-diffracting crystals, x-ray doses within the acceptable room temperature limit yield datasets that compare well to their XFEL counterparts.^137^ At moderate repetition rates, ranging from 60 to 120 Hz as employed by Pohang accelerator laboratory (PAL)-XFEL, Spring-8 Ångstrom compact free electron laser (SACLA), SwissFEL, and LCLS, serial crystallography proves exceptionally efficient. The European XFEL and LCLS2 are pushing the envelope with megahertz repetition rates, although these present challenges for time-resolved experiments.^85^ Repetition rates around 1000 Hz appear to align better with current sample delivery and detector capabilities. XFELs with such repetition rates could extend their lead over synchrotrons beyond ultrafast experiments due to an increase in throughput.

Already at third-generation synchrotrons, time-resolved serial crystallographic studies were carried out and PETRA III even has the first dedicated endstation for time-resolved crystallographic studies (T-REXX), leading the first wave of time-resolved ssSX experiments at synchrotrons.^81,83^ Overlaps exist among third- and fourth-generation synchrotrons and XFELs, with the choice depending on the experiment. Third-generation synchrotrons are suited to study well-diffracting crystals in milliseconds, while fourth-generation synchrotrons are better for microsecond studies due to increased flux densities. XFELs excel in studies below nanoseconds and minimize site-specific radiation damage, especially in metalloproteins. Since synchrotrons can counter radiation damage only by distributing the dose across a larger number of crystals, measurement time is an important factor in time-resolved experiments, a key advantage of the emerging fourth-generation sources. These new sources, coupled with improvements in signal-to-noise ratios, are expected to narrow the resolution gap between synchrotrons and XFELs for many crystal systems, except perhaps for very small crystals^136^ or crystals having a large unit cell.

The latest fourth-generation synchrotron sources, with endstations focusing on serial crystallography, are ideally positioned for time-resolved experiments. Facilities like MaxIV, National Synchrotron Light Source (NSLS-2), and European Synchrotron Radiation Facility (ESRF) have already upgraded, with Advanced Photon Source (APS) and Swiss Light Source (SLS-2) soon to follow.^120^ There is a growing emphasis on pink-beam serial crystallography^138,139^ using multi-layer monochromators at beamlines such as ESRF's ID 29 and the upcoming MicroMax at MaxIV, specifically tailored for time-resolved studies. The pink beam, with its exceptionally high photon flux, can deliver the full maximum dose to a crystal in microseconds, increasing dataset convergence rates due to its ability to excite multiple reflections simultaneously, albeit at the expense of signal-to-noise and increased damage per diffracted photon. This technique effectively transforms synchrotrons into high repetition rate XFELs, capable of collecting microsecond time-resolution data rapidly, albeit without the benefit of diffraction-before-destruction. In combination with spectroscopic studies on crystals,^140^ these “pulsed” sources enable selective targeting of kinetically stable structural intermediates. Given that many current time-resolved studies show only small rearrangements in the nanosecond or faster temporal regimes, microsecond time resolution appears sufficient to capture most biologically relevant changes.

**FIG. 4.**
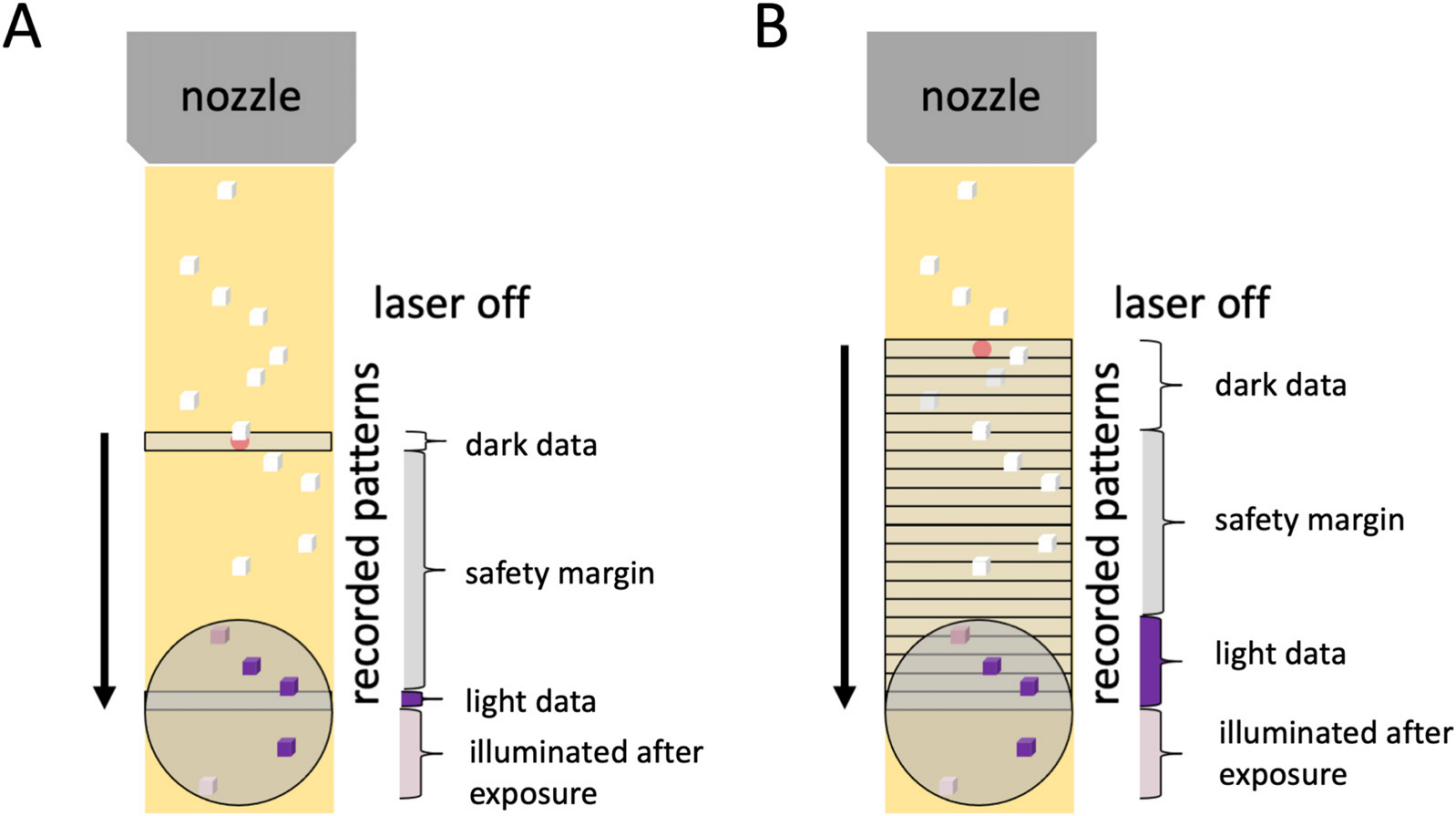
Variants of time-resolved single-shot serial crystallography (ssSX) via a high-viscosity jet. (a) The pump-probe method activates crystals (depicted as purple blocks) with a laser pulse (gray circle), with the jet (yellow) extrusion allowing collection from non-illuminated crystals (white blocks). Data acquisition follows with an x-ray pulse for a single diffraction pattern at a set time delay, then a dark pattern once the jet moves the activated zone past the laser's effective diameter (at least 1.5 times the 1/e^2^ size of the pump laser). This mode is suitable when the probe pulse dose exceeds the damage threshold, a scenario well-characterized at XFELs but requiring rigorous assessment at fourth-generation synchrotrons, especially when utilizing multi-layer monochromators that deliver high doses in short durations. (b) The pump-scan technique^81^ continuously probes the illuminated region, compiling diffraction patterns over a duration until the pumped area is displaced by jet extrusion, thus capturing an entire time-series rather than a fixed delay. This approach is feasible when the dose per frame remains within the radiation damage threshold of about 100 kGy per frame.

Another approach that is uniquely suited to synchrotron sources and can be implemented at virtually all third- and fourth-generation synchrotron sources allows a very efficient dose distribution and increased sample utilization: collecting entire time-series at once in a pump-scan approach.^81^ Very fast data collection at fourth-generation synchrotrons^141^ will render this method highly efficient, potentially increasing throughput by an order of magnitude. With the aid of fast detectors, reactions can be initiated across large sample volumes, and the ensuing time-resolved changes monitored, while mitigating radiation damage by translating the sample through the beam (Fig. 4). The vast number of diffraction patterns collected in serial crystallography result in highly uniform datasets, simplifying the comparison between individual timepoints. This homogeneity may facilitate precise tracking of the rise and decay of structural intermediates, deconvolution of overlapping states and allow approaching nanosecond time resolutions when using techniques like the Hadamard transform^133^ and gateable detectors.

## TRIGGERING TIME-RESOLVED INSIGHTS: FROM LASERS TO LIGANDS

**FIG. 5.**
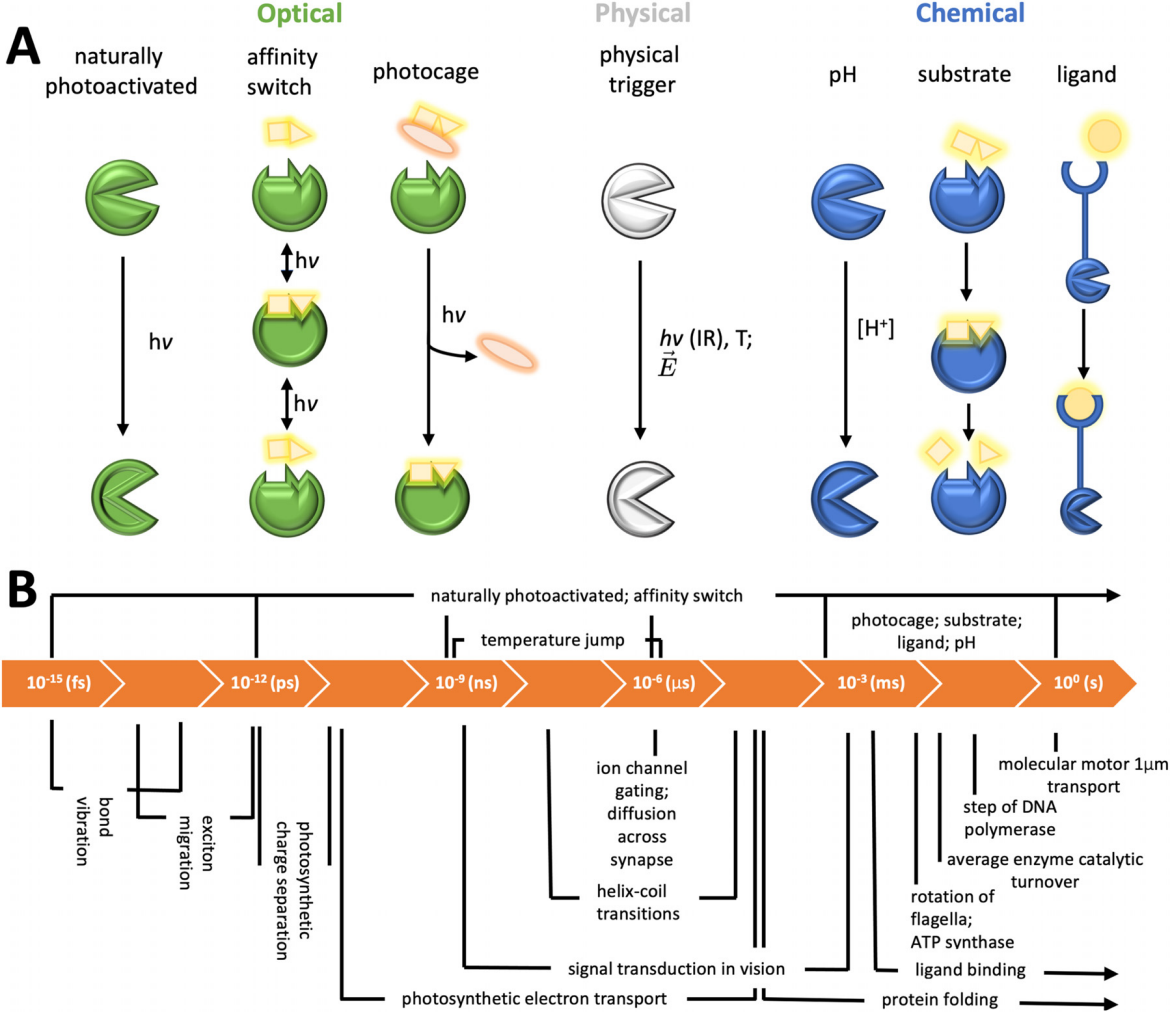
Overview of triggering mechanisms and corresponding biological processes in time-resolved crystallography. (a) Classification of triggering mechanisms by the nature of the initiating stimuli, with the corresponding time scales increasing from left to right. (b) The time-scales of biologically relevant processes^151^ and their alignment with the triggering mechanisms depicted in (a), illustrating the timescale coverage for capturing dynamic biological events.

With the advent of high-speed detectors and serial crystallography techniques that distribute x-ray exposure across multiple crystals, the structural biology toolkit for studying protein dynamics is now fully equipped. More optimized endstations at both fourth-generation synchrotrons and XFELs have integrated the latest technologies and are making them accessible to the structural biology community. The focus of time-resolved crystallography now lies on manipulating the biochemical system to trigger meaningful reactions (Fig. 5).

Processes that can be triggered by laser light offer the opportunity to capture successive snapshots of protein activation, elucidating structural changes on the levels of individual atoms.^142^ Light-activated proteins have evolved to harness light as an energy source. Therefore, although they constitute a small fraction of all proteins,^143^ they offer a unique view of protein function across all timescales.

In the absence of a natural light trigger, it is possible to use chemical reactions triggered by light. Photocages, which possess photolabile groups, release active ligands or enzyme substrates upon laser activation.^143^ Although photocages often do not bind directly to proteins, the subsequent binding of the released compound can be monitored,^76^ typically achieving millisecond time resolutions. While this timescale may not allow observation of rapid side chain conformational changes, which usually occur within nanoseconds to microseconds,^43,89^ it is suitable for detecting larger structural shifts^81^ and monitor enzyme catalysis.^82^

Photochemical affinity switches offer another means to utilize laser light to induce a protein response. Designed as photopharmacological compounds for key drug targets, these molecules can be toggled by light between low and high affinity states, potentially allowing more targeted drug activation within specific tissues. By inducing a low affinity state in a ligand-protein complex, one can track the binding pocket's adaptation and subsequent ligand dissociation across extensive temporal ranges,^110,144^ but also ligand binding experiments may be possible using the method.

Beyond photochemical triggers, physical triggers like infrared (IR) laser induced temperature jumps (T-jumps) are employed to study protein dynamics.^145^ This method rapidly heats samples using IR lasers, primarily transferring energy to water molecules' vibrational modes, which then quickly dissipates to other molecules, raising the sample temperature almost instantaneously. T-jumps have been utilized to probe conformational states during enzyme catalysis and can be applied within protein crystals to induce active conformations, as demonstrated in time-resolved wide-angle x-ray scattering (WAXS) experiments^146^ and serial crystallography.^116^ Another example for a physical trigger that has been used in crystallography is electric field stimulation.^71^

Rapid mixing techniques at XFELs and synchrotrons enable the observation of chemical reactions in real-time by combining substrates with crystals. For micrometer-sized crystals of many enzymes that have turnover rates about ten per second or less, diffusion times are negligible, allowing for immediate interaction with enzymes, provided the first reaction step is slow enough to reach a discernible starting point, preventing electron density blurring. That said, particularly bulky and hydrophobic ligands can be very slow to diffuse, and it is known that protein crystals can form a skin of denatured protein which makes them more stable, but interferes with ligand soaking. However, successful mixing experiments have shown ligand binding to targets within the early milliseconds,^83,102^ a timeframe adequate for studying many enzymatic reactions and possibly larger conformational changes in signaling processes, if the crystal system accommodates them.

## MOLECULAR MOVIES: VISUALIZING PROTEIN DYNAMICS

Crystallography, while unmatched in capturing high-resolution structural data, relies on observing large populations of highly ordered molecules. Consequently, it predominantly reveals highly populated states.^147^ Examining transitions between these states at various temperatures can provide insights into kinetic barriers and the nature of transitions.^148^ However, the actual atomic motions often occur at such rapid timescales that they are only observable in non-equilibrium processes triggered and synchronized by femtosecond laser pulses and, hence, must be probed in the same timescale.^11^ The advent of XFELs has enabled crystallography to investigate atoms outside the Boltzmann distribution, leading to breakthroughs in understanding processes like the cis-trans isomerization in the photoactive yellow protein chromophore,^26^ the dissociation of carbon monoxide from myoglobin,^25^ and the isomerization of retinal in bacteriorhodopsin^77^ as well as the photoreaction of azobenzene,^144^ leading to true molecular movies of atom ensembles in motion.

To further refine time-resolved studies, computational approaches may enhance the temporal resolution of XFEL experiments.^149^ These methods may outperform the binning-and-merging of ultrafast data since data sparsity is mitigated in coherent time-resolved crystallographic experiments.^150^ However, their application might be constrained to the sub-picosecond regime due to the necessity of continuous structural evolution. While interpolating kinetically stable structural intermediates may be biophysically simplistic, the didactic value in summarizing study findings is significant. These visual synopses often dubbed “molecular movies” elucidate structural dynamics and offer a more comprehensive mechanistic interpretation than single static structures obtained from frozen proteins.

Computational simulations excel in modeling chemical events like bond formation, starting from accurate structural coordinates. For instance, in studying enzyme catalysis, the short-lived transition state is best approached via simulation, provided there are precise intermediate structures resolved by dynamic crystallography. Meanwhile, structural intermediates occurring on millisecond timescales might elude computational methods. Therefore, capturing high-resolution intermediates along a reaction pathway and linking them through molecular dynamics simulations is emerging as an effective strategy to integrate the insights of time-resolved serial crystallography with quantum mechanics and molecular mechanics simulations. This synergy allows for the creation of molecular movies that narrate the full story of protein dynamics and function.

## CONCLUSION: CRYSTALLOGRAPHY'S DYNAMIC FUTURE

Moffat and colleagues made the first step toward capturing protein structural intermediates through crystallography.^13^ Petsko urged the time-resolved community to focus on the scientific questions that can be addressed rather than only expanding the method,^10^ a challenge his postdoc Schlichting rose to meet.^17^ While a PostDoc with Hajdu, Neutze showed that the problem of radiation damage can be overcome by the use of femtosecond x-ray pulses.^122^ A decade later, the advent of serial crystallography at XFELs by Chapman, Spence, and their teams made good on this promise.^39^ Shortly after, the groups of Schmidt, Schlichting, and Neutze showcased that serial crystallography and XFELs could indeed bring time-resolved crystallography into the mainstream.^25,27,43^

Presently, with over 150 000 structural templates available for dynamic studies in the wwPDB,^152^ and with the major hurdles of radiation damage and rapid data acquisition addressed, time-resolved crystallography is poised to explore almost any crystallizable system. As the structural biology field gravitates toward cryo-EM,^153^ synchrotrons are reallocating beamtime to time-resolved crystallography, with specialized beamlines emerging globally at next-generation sources.

The structural dynamics community is now positioned to leverage this beamtime to elucidate nearly damage-free structures at physiological temperatures. Using current structural knowledge, ideal experimental conditions can be pinpointed, and appropriate methods can be selected to shed light on protein function. Advanced mutagenesis techniques can be applied to dynamic studies of functional mutants, and temperature variations can be used to derive kinetic insights and distinguish between states.^148^

The influx of data from these diverse experiments will necessitate and drive the evolution of crystallographic software which has already begun,^154–156^ to fully harness the additional dimensions that time-resolved studies introduce into crystallography.

The principle of understanding function through structure remains as relevant as ever, underscored by the triumphs of cryo-EM and AlphaFold. However, crystallography now stands on the threshold of directly observing function, a capability that promises to be as revolutionary as structural biology itself. Integrating these data into a structural dynamics database could catalyze the development of machine learning-based computational dynamic methods, potentially making the prediction of dynamic protein motions as routine as protein folding prediction is today.

## Data Availability

All data used to generate figures are publicly available via the wwPDB.
